# Ti_0.87_O_2_ Nanosheet-Associated Aramid Nanofiber Ordering in Ultrathin Membranes for Proton Gradient Energy Conversion

**DOI:** 10.3390/nano16181149

**Published:** 2026-09-14

**Authors:** Xinyue Qu, Zhongxi Long, Ling Qiu

**Affiliations:** Shenzhen Geim Graphene Center (SGC), Tsinghua Shenzhen International Graduate School, Tsinghua University, Shenzhen 518055, China; longzx24@mails.tsinghua.edu.cn (Z.L.); ling.qiu@sz.tsinghua.edu.cn (L.Q.)

**Keywords:** aramid nanofibers, Ti_0.87_O_2_ nanosheets, proton transport, polymer ordering, ion-selective membranes, reverse electrodialysis

## Abstract

In membrane-based reverse electrodialysis (RED), ion-selective membranes convert ionic concentration gradients directly into electrical output. Protons are often treated within the general framework of cation selectivity, while how membrane structure specifically regulates proton transport beyond conventional cation transport remains insufficiently understood. Here, freestanding aramid nanofiber (ANF)/Ti_0.87_O_2_ membranes approximately 3 μm thick are prepared by co-dispersion in trifluoromethanesulfonic acid (TfOH), spin coating, aqueous regeneration, and confined drying. X-ray diffraction and two-dimensional wide-angle X-ray scattering show that Ti_0.87_O_2_ incorporation is accompanied by enhanced ANF ordering and preferential orientation. After normalization to the bulk HCl/KCl conductivity ratio, the proton preference factor increases from approximately 1.0 for ANF to 1.67 at a Ti_0.87_O_2_ mass fraction of 0.5, while the apparent activation energy is lower for HCl than for KCl transport (0.18 versus 0.24 eV). Under a 1000-fold HCl concentration gradient (1 M/0.001 M), the membrane delivers 51.52 W m^−2^ and retains 88.1% of its short-circuit current over approximately 33 h. Further integration into a 10-unit stack yields an open-circuit voltage of 1.77 V, demonstrating modular voltage scaling. Together, these results suggest that Ti_0.87_O_2_ nanosheets promote ANF ordering and contribute to proton-favorable transport behavior.

## 1. Introduction

Selective ion transport is central to membrane processes for energy conversion, separation, and electrochemical systems [1,2,3,4]. Salinity-gradient energy can be harvested through membrane-based technologies such as pressure-retarded osmosis (PRO) and reverse electrodialysis (RED) [5]. PRO converts osmotically driven water transport into hydraulic work, whereas RED directly converts selective ion transport into electrical potential and current. In nanostructured membranes, ion selectivity is determined not only by pore dimensions and surface charge, but also by the organization, continuity, and local chemical environment of transport pathways [6,7]. Controlling molecular and nanoscale organization within membranes therefore offers an important route to regulate ion transport beyond conventional control through size exclusion or electrostatic interactions [8,9]. However, establishing clear relationships between membrane organization and the preferential transport of specific ions remains challenging because structural effects are often coupled with hydration, swelling, charge distribution, and overall permeability [10,11]. In particular, interfacial chemistry, pH-dependent protonation, and nanoconfinement can reorganize confined water and its hydrogen bond dynamics, thereby modifying the local hydration environment relevant to ion transport [12,13].

Protons are distinct from conventional hydrated cations because their transport in water can involve both molecular diffusion and structural diffusion through hydrogen-bonded networks, resulting in exceptionally high mobility [14,15]. This distinctive transport behavior has motivated membrane systems for harvesting energy from proton concentration gradients. Acidic industrial streams can provide chemical-potential gradients that may be exploited through different membrane-based routes. PRO has been explored for osmotic power generation using acidic or acid-conditioned wastewater streams. For example, a recent PRO study using an acidic mining effluent (pH 3.8) reported a gross power density of 18.6 W m^−2^ [16], while acidification of municipal reverse osmosis retentate to pH 5.5 increased the PRO power density from 7.3 to 12.6 W m^−2^ by mitigating membrane fouling [17]. In contrast, proton gradient RED directly exploits selective proton transport across ion-selective membranes [18,19]. Aramid nanofiber (ANF) membranes produced an average output power density of 17.3 W m^−2^ under strongly acidic conditions, while Ti_3_C_2_T_x_ MXene membranes delivered 6.5 W m^−2^ under a 1000-fold HCl concentration gradient [20,21]. More recently, framework membranes and designed proton channels have been developed to improve transport under strongly acidic conditions [22,23,24,25,26]. Notably, linkage-encoded covalent organic framework (COF) membranes recently achieved a peak osmotic power density of 2422.9 W m^−2^ under a 12 M/0.01 M H_2_SO_4_ gradient with an effective membrane area of 0.03 mm^2^ [26], illustrating the exceptionally high membrane-level output achievable under concentrated-acid conditions. Despite the high membrane-level power densities reported in laboratory-scale systems, practical scale-up remains challenging. Increasing membrane area and device size can introduce additional transport resistance, concentration polarization, hydraulic losses, and auxiliary energy consumption, such that high area-normalized power density does not necessarily translate into proportionally high total or net power output. Commercial Nafion membranes generated 5.1 W m^−2^ over a test area of 0.2 mm^2^, whereas the power density decreased to 2.1 W m^−2^ when the test area was increased to 12.5 mm^2^ [27]. These studies have mainly regulated proton transport through channel dimensions, charge, functional groups, and confined ionic environments [28,29]. However, whether the structural organization of the membrane framework itself, particularly the ordering of a rigid polymer framework, can generate a specific preference for proton transport remains much less explored.

Rigid polymer frameworks are particularly suitable for examining this question because their chain organization can be regulated while maintaining a mechanically stable membrane structure [30,31]. This effect may be particularly relevant to proton transport, because proton transfer depends on hydrogen-bonded environments that are sensitive to local interfacial chemistry and hydration [32,33]. Aramid nanofibers, composed of rigid poly(p phenylene terephthalamide) chains, provide a mechanically robust framework whose molecular organization can be regulated during membrane formation [34,35,36]. Ti_0.87_O_2_ nanosheets provide extended two-dimensional Ti-deficient oxide interfaces [37] with proton-responsive surface chemistry [38]. A previous study combined Ti_0.87_O_2_ nanosheets with ANF in lamellar membranes for osmotic energy conversion, primarily emphasizing confined ion transport and ion selectivity [39]. However, whether Ti_0.87_O_2_ incorporation can regulate ANF organization, and whether the resulting structural ordering specifically favors proton transport beyond the intrinsic mobility of H^+^ in water, remains unclear.

Here, we develop a reverse electrodialysis (RED)-type proton gradient energy conversion system based on freestanding ANF/Ti_0.87_O_2_ membranes. ANF/Ti_0.87_O_2_ membranes approximately 3 μm thick were prepared by co-dispersion of both components in trifluoromethanesulfonic acid (TfOH), followed by spin coating, regeneration in water, and confined drying. X-ray photoelectron spectroscopy (XPS) and Fourier-transform infrared spectroscopy (FTIR) identify changes in the local amide interaction environment, while powder X-ray diffraction (XRD) and two-dimensional wide-angle X-ray scattering (2D WAXS) show that Ti_0.87_O_2_ incorporation is accompanied by substantially enhanced ANF ordering and preferential orientation. We then evaluate proton preference associated with the membrane architecture by normalizing HCl/KCl transport against the corresponding bulk conductivity ratio and comparing their apparent activation energies. Finally, proton gradient energy conversion is evaluated in a RED-type configuration, followed by multi-unit membrane integration to assess the scalability of the electrical output. Through these analyses, we examine whether Ti_0.87_O_2_-associated ANF organization is correlated with proton-preferential transport beyond the intrinsic mobility advantage of H^+^ in bulk water.

## 2. Materials and Methods

### 2.1. Materials

Kevlar chopped fibers (DuPont, Kevlar^®^ K29, Wilmington, DE, USA), trifluoromethanesulfonic acid (TfOH; Aladdin, Shanghai, China; ≥99.5%), TiO_2_ (Aladdin; ≥99.9%), K_2_CO_3_ (Aladdin; ≥99%), Li_2_CO_3_ (Aladdin; ≥99.99%), tetra-*n*-butylammonium hydroxide (TBAOH; Aladdin; ~25% in H_2_O), hydrochloric acid (HCl; Guangzhou Chemical Reagent Factory, Guangzhou, China; 36–38%), KCl (Aladdin; ≥99.8%), NaCl (Aladdin; ≥99.8%), LiCl (Aladdin; ≥98%), CaCl_2_ (Aladdin; ≥97%), and MgCl_2_ (Aladdin; ≥98%) were used. PiperION^®^ A20 anion exchange membranes were from Versogen (Newark, DE, USA). Deionized water (18.2 MΩ·cm) was used for membrane preparation and electrolyte solutions.

### 2.2. Preparation of Ti_0.87_O_2_ Nanosheets

Ti_0.87_O_2_ nanosheets were prepared from layered K_0.8_Ti_1.73_Li_0.27_O_4_. TiO_2_, K_2_CO_3_, and Li_2_CO_3_ were mixed at a molar ratio of 1.73:0.40:0.14 and calcined at 900 °C for 20 h. The precursor was stirred in 0.5 M HCl at room temperature for 48 h to form H_1.07_Ti_1.73_O_4_·H_2_O, then filtered, washed, and air-dried. The protonated titanate was exfoliated with aqueous tetra-*n*-butylammonium hydroxide (TBAOH) at a TBA^+^/H^+^ molar ratio of 1:1 by shaking for 7 days. The resulting Ti_0.87_O_2_ nanosheet suspension was freeze dried.

### 2.3. Preparation of ANF/Ti_0.87_O_2_ Membranes

Kevlar chopped fibers were dispersed in TfOH at an ANF concentration of 15 mg mL^−1^. Ti_0.87_O_2_ solids were added at the designed mass ratio and dispersed to obtain a homogeneous ANF/Ti_0.87_O_2_ dispersion. Unless otherwise specified, the ANF:Ti_0.87_O_2_ mass ratio was 1:1. For the composition-dependent samples, the Ti_0.87_O_2_:ANF mass ratios were 1:3 and 2:3. The dispersion was spin coated onto a silicon substrate at 1000 rpm for 20 s. The coated film was immersed in deionized water for 1 h for aqueous regeneration, solvent exchange, and TfOH removal. The hydrated membrane was then vacuum dried under confinement at 40 °C for 24 h and released as a freestanding membrane.

ANF control membranes were prepared from the 15 mg mL^−1^ ANF/TfOH dispersion without Ti_0.87_O_2,_ using the same spin coating, water regeneration, and confined drying conditions.

### 2.4. Characterization

Scanning electron microscopy (SEM) images of exfoliated Ti_0.87_O_2_ nanosheets and membrane cross-sections were acquired using a Hitachi SU8010 microscope (Hitachi High-Tech Corporation, Tokyo, Japan), and cross-sectional elemental distributions were examined by energy-dispersive X-ray spectroscopy (EDS). Atomic force microscopy (AFM) images of the nanosheets were recorded with a Bruker Dimension Icon system (Bruker Nano Surfaces and Metrology, Santa Barbara, CA, USA).

X-ray photoelectron spectroscopy (XPS) spectra of Ti_0.87_O_2_, ANF, and the ANF/Ti_0.87_O_2_ membrane were recorded using a Thermo Scientific K-Alpha system (Thermo Fisher Scientific, East Grinstead, UK) with Al Kα radiation. The C 1s spectrum was fitted first; the C–C/C=C component was referenced to 284.8 eV, and the same correction was applied to the corresponding high-resolution spectra.

Fourier-transform infrared spectroscopy (FTIR) spectra were collected in attenuated total reflectance (ATR) mode over 4000–400 cm^−1^ using a PerkinElmer Spectrum 3 spectrometer (PerkinElmer, Shelton, CT, USA); dry and wet membranes were also compared. X-ray diffraction (XRD) patterns were recorded with a Bruker D8 ADVANCE (Bruker AXS GmbH, Karlsruhe, Germany) diffractometer using Cu Kα radiation over approximately 2θ = 5–40°. For wet-state XRD, the ANF/Ti_0.87_O_2_ membrane was immersed in deionized water for 1 h and excess surface water was removed before measurement.

2D WAXS patterns of ANF and ANF/Ti_0.87_O_2_ membranes were collected using a Xeuss 2.0 system (Xenocs, Grenoble, France) with Cu Kα radiation, with the X-ray beam incident perpendicular to the membrane plane. Radial intensity profiles and azimuthal profiles of relevant reflections were extracted to evaluate the preferred orientation.

The surface zeta potential of the ANF/Ti_0.87_O_2_ membrane was measured as a function of pH using a SurPASS 3 analyzer (Instron, Norwood, MA, USA) in streaming potential mode. Uniaxial tensile tests were performed using an Instron 5943 system.

### 2.5. Ion-Transport Measurements

ANF/Ti_0.87_O_2_ membranes were mounted between the two chambers of a custom electrochemical cell with an effective area of 0.03 mm^2^. Homemade Ag/AgCl electrodes directly contacted the solutions, whereas commercial Ag/AgCl electrodes with a single salt bridge (Gaoss Union, Wuhan, China) were used to minimize the electrode redox contribution. A Bio-Logic SP-200 (BioLogic Science Instruments, Seyssinet-Pariset, France) applied the transmembrane voltage and recorded current. Symmetric measurements used HCl at 0.001, 0.01, 0.1, or 1 M in both chambers; 0.1 M HCl and KCl were compared. Voltage was scanned from −0.3 to 0.3 V at 10 mV s^−1^ after 5 s equilibration. Conductance, *G*, was obtained from the linear-region slope of each linear sweep voltammetry (LSV) current–voltage (*I*–*V*) curve. All ion transport measurements were repeated independently three times, and the averaged values were used for data analysis. Data are presented as mean ± standard deviation from three independent measurements.

LSV measurements at 25, 35, 45, 55, and 65 °C were conducted in symmetric 0.1 M HCl and KCl using a thermostatic water bath after 30 min equilibration. The apparent activation energy was determined by fitting ln *G* versus 1000/*T* using the Arrhenius relationship.

$$\ln G=\ln G_{0}-E_{a}/RT$$

where *G*_0_ is the pre-exponential factor, *E*_a_ is the apparent activation energy, *R* is the gas constant, and *T* is the absolute temperature.

Bulk HCl and KCl conductances were measured using the same cell geometry to provide the reference ratio used for normalization.

For concentration gradient measurements, *C*_L_ was fixed at 0.001 M for HCl, and *C*_H_/*C*_L_ was 10, 50, 100, 500, or 1,000. HCl and KCl were compared at a concentration ratio of 1000. Additional measurements at this ratio used HCl, KCl, NaCl, LiCl, CaCl_2_, and MgCl_2_ at 1 M/0.001 M.

With Ag/AgCl electrodes in direct contact with the solutions, the raw open circuit voltage, *V*_OC_, comprised the membrane diffusion potential, *V*_diff_, and the electrode redox potential difference, *V*_redox_, which was caused by unequal chloride activities. Ag/AgCl electrodes isolated by salt bridges were used to minimize this contribution. Corrected *V*_diff_ and diffusion current, *I*_diff_, were the voltage and current axis intercepts, respectively, of the corrected *I*–*V* curve.

The cation transference number, *t*_+_, was calculated from the corrected diffusion potential using the Planck–Henderson diffusion potential relationship [40,41,42] for a binary 1:1 electrolyte:

$$t_{+}=\;1/2\;\left[1+zF\left|V_{diff}\right|/\left(RT\;\ln\left(a_{H}/a_{L}\right)\right)\right]$$

where *t*_+_ + *t*_−_ = 1, *z* = 1 for HCl, *R* is the universal gas constant, *F* is the Faraday constant, and *T* is the absolute temperature. *a*_H_ and *a*_L_ are the mean ionic activities [43] on the high and low concentration sides, respectively, calculated as *a*_H_ = *γ*_H_ *C*_H_ and *a*_L_ = *γ*_L_ *C*_L_, where *γ* is the mean ionic activity coefficient. Activity coefficients and calculated values are provided in Table S1.

### 2.6. RED-Type Proton Gradient Energy Measurements and Device Integration

Single-membrane RED-type proton gradient power measurements used Ag/AgCl electrodes isolated by salt bridges and HCl concentration ratios of 10, 100, and 1000, with *C*_L_ = 0.001 M. An external load resistance, *R*_L_, was connected, with current, *I*, recorded at each resistance. Power density, *P*, was calculated as

$$P=I^{2}R_{L}/A$$

where *I* is the load current, *R*_L_ is the external load resistance, and *A* is the effective membrane area (0.03 mm^2^). Load-dependent power measurements were repeated three times under identical conditions, and the averaged values are reported. The short circuit current stability was evaluated separately under a 1 M/0.001 M HCl gradient for approximately 33 h. The two electrolyte reservoirs had a volume of 500 mL each and were continuously circulated using peristaltic pumps at 20 mL min^−1^ to minimize concentration changes during measurement.

Integrated devices comprised alternating ANF/Ti_0.87_O_2_ and PiperION^®^ A20 anion exchange membranes between end plates, flow-channel plates, and seals. Adjacent channels contained 1 M and 0.001 M HCl. Membrane orientations ensured additive potentials. The effective area was 3 mm^2^. Up to 10 units were tested by recording open circuit voltage and short circuit current.

## 3. Results and Discussion

### 3.1. Fabrication and Morphology of ANF/Ti_0.87_O_2_ Membranes

**Figure 1:**
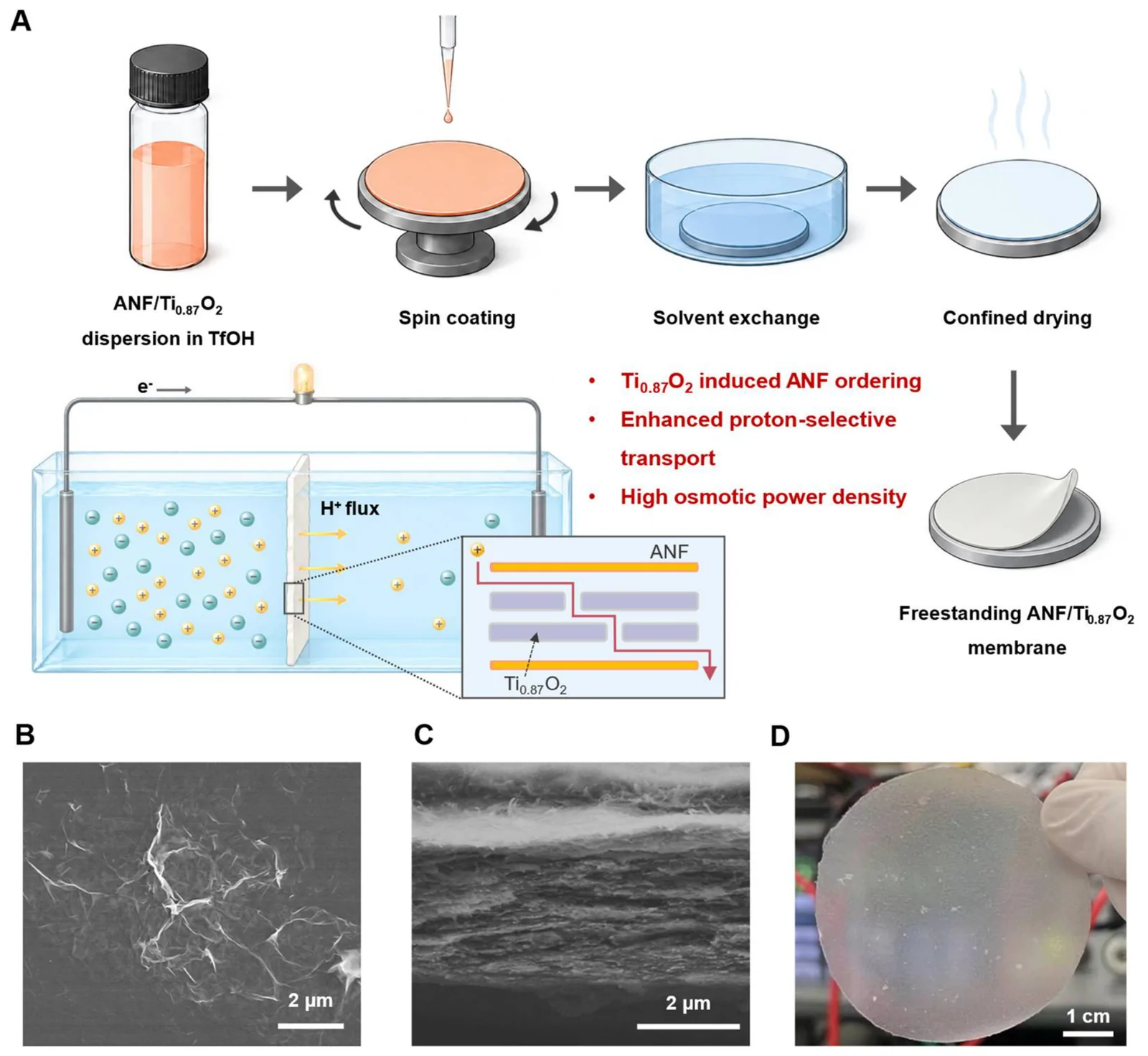
Fabrication and morphology of freestanding ANF/Ti_0.87_O_2_ membranes. (**A**) Schematic of membrane fabrication and RED-type proton gradient energy conversion. In (**A**), the curved black arrows indicate substrate rotation during spin coating, while the pink arrow schematically indicates the direction of proton transport across the membrane. (**B**) SEM image of exfoliated Ti_0.87_O_2_ nanosheets. (**C**) Cross-sectional SEM image of the ANF/Ti_0.87_O_2_ membrane. (**D**) Photograph of a freestanding membrane.

Freestanding ANF/Ti_0.87_O_2_ membranes were fabricated from exfoliated Ti_0.87_O_2_ nanosheets [44,45,46] and a 15 mg mL^−1^ ANF/TfOH dispersion [47] by co-dispersion, spin-coating, aqueous regeneration, and confined drying (Figure 1A; Section 2.3 and Figure S1). Unless otherwise noted, the ANF:Ti_0.87_O_2_ mass ratio was 1:1.

Scanning electron microscopy (SEM) shows the ultrathin, sheet-like morphology of the exfoliated Ti_0.87_O_2_ material (Figure 1B). Atomic force microscopy imaging and the corresponding thickness and lateral size statistics (Figure S2A–C) gives an average thickness of 1.157 nm and an average lateral size of 1.603 μm, consistent with effective exfoliation. The composite membrane has a thickness of approximately 3.0 μm and a compact lamellar cross-section without visible macroscopic voids (Figure 1C). Cross-sectional EDS mapping further shows Ti distributed across the mapped membrane cross-section together with N from the ANF phase, without obvious micron-scale Ti-rich aggregation (Figure S2D–F), supporting the formation of a spatially integrated ANF/Ti_0_._87_O_2_ composite architecture. The macroscopic membrane is uniform, flexible, and self-supporting (Figure 1D). These observations establish a dense membrane platform for changing the organization of the ANF matrix.

### 3.2. Ti_0.87_O_2_-Associated Aramid Ordering

**Figure 2:**
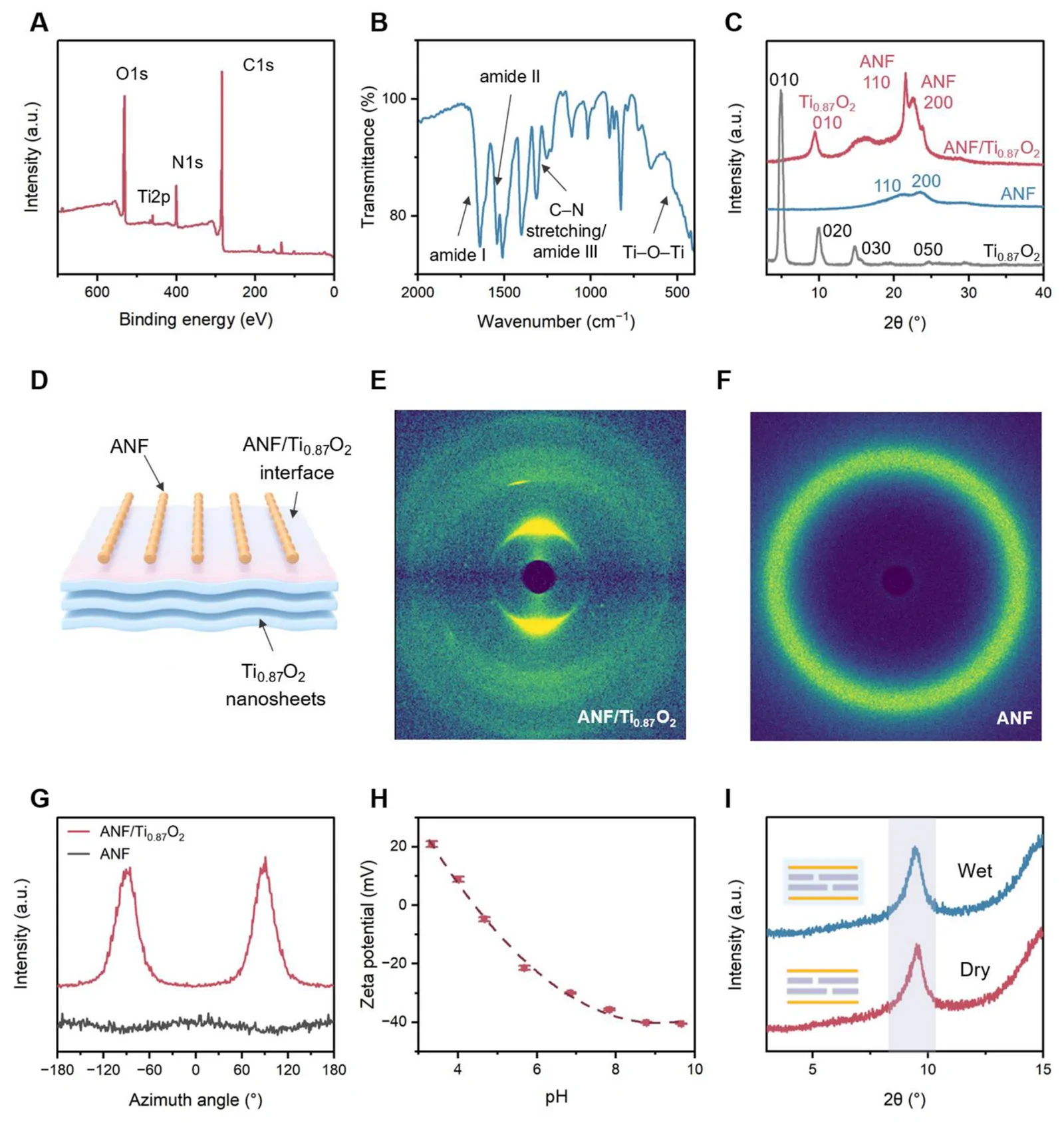
Ti_0.87_O_2_-associated aramid ordering. (**A**) XPS survey spectrum and (**B**) FTIR spectrum of the ANF/Ti_0.87_O_2_ membrane. (**C**) XRD patterns of restacked Ti_0.87_O_2_, the ANF membrane, and the ANF/Ti_0.87_O_2_ membrane. (**D**) Schematic of the ordered ANF/Ti_0.87_O_2_ interfacial architecture. 2D WAXS patterns of (**E**) the ANF/Ti_0.87_O_2_ membrane and (**F**) the ANF membrane. (**G**) Corresponding azimuthal intensity profiles. (**H**) Streaming-potential-derived zeta potential of the ANF/Ti_0.87_O_2_ membrane as a function of pH. (**I**) XRD comparison of the membrane before and after water immersion. The schematic insets depict the lamellar architecture in the dry and wet states, with orange lines and purple blocks representing ANFs and Ti_0.87_O_2_ nanosheets, respectively.

XPS and FTIR were first used to examine the local chemical environment of the ANF matrix after nanosheet incorporation. The XPS survey spectrum contains the Ti 2p signal together with the O 1s, N 1s, and C 1s signals expected for the composite (Figure 2A). Additional XPS survey spectra and fitted Ti 2p and N 1s spectra (Figure S3) show that the Ti–O framework remains the dominant Ti chemical environment, although the Ti signal is attenuated by the polymer rich surface. Relative to the ANF membrane, the fitted N 1s component shifts modestly from 400.25 to 399.98 eV in the composite. This shift is consistent with a modified local amide interaction environment rather than the formation of a new covalent bond.

The FTIR spectrum retains the characteristic amide I, amide II, and C–N stretching/amide III bands of ANF together with the Ti–O–Ti response of Ti_0.87_O_2_ (Figure 2B). Measurements on three separately prepared membranes of each type show that the N–H stretching band shifts from 3302.65 ± 1.07 cm^−1^ for ANF to 3317.91 ± 1.42 cm^−1^ for the composite, while the amide I band shifts from 1637.63 ± 2.50 to 1642.60 ± 1.27 cm^−1^ (mean ± standard deviation, *n* = 3; Figure S4). Expanded spectra and peak-position statistics confirm that the shifts toward higher wavenumbers recur across membrane samples. The combined spectral changes are consistent with reorganization toward a more homogeneous local amide and hydrogen bond environment [48]. Such relaxation of interchain hydrogen bond constraints may increase the ability of ANF building blocks to rearrange and orient during membrane processing [36,49]. Thus, XPS and FTIR are consistent with an altered local ANF/Ti_0.87_O_2_ interfacial environment while the principal chemical identities of both components are preserved.

XRD provides direct evidence that this interfacial reconstruction is accompanied by a pronounced change in ANF organization (Figure 2C). The restacked Ti_0.87_O_2_ solid displays the expected (010), (020), (030), and (050) reflections, whereas the ANF membrane shows only weak ANF (110) and (200) features. In the composite, the Ti_0.87_O_2_ (010) reflection shifts to 2θ = 9.42°, consistent with a contracted restacked nanosheet spacing [39]. More importantly, the ANF-related reflections become substantially more pronounced [50,51]. Peak fitting assigns the features at 21.55° and 22.41° to the ANF (110) and (200) reflections, with full widths at half maximum (FWHM) of 0.35° and 2.52°, respectively; the fitted ANF-related contribution reaches 27.18% of the integrated area in this region. These changes demonstrate enhanced ANF chain packing and structural ordering. The corresponding interfacial architecture is illustrated schematically in Figure 2D. Because diffraction intensity can also be amplified by texture, the orientation component was evaluated independently by 2D WAXS.

The ANF control produces a nearly continuous, weakly anisotropic scattering ring, whereas the ANF/Ti_0.87_O_2_ membrane displays concentrated arcs (Figure 2E,F). With the incident beam normal to the membrane plane, the pronounced azimuthal maxima in Figure 2G demonstrate preferential in-plane orientation in the composite, while the ANF control shows little azimuthal modulation. The ANF-related and nanosheet-related anisotropies follow the same principal direction, supporting correlated organization of the rigid polymer chains and two-dimensional sheets. Together, the XRD and WAXS results establish substantially enhanced ANF ordering and preferential orientation.

Streaming-potential measurements on the ANF/Ti_0.87_O_2_ membrane show that its electrokinetic zeta potential decreases from approximately +20 mV under acidic conditions to approximately −40 mV under alkaline conditions, with an apparent isoelectric point near pH 4.5 (Figure 2H). This pH-dependent response is consistent with reversible protonation and deprotonation of interfacial groups. Because the zeta potential is defined at the slipping plane under the analyzer test conditions, its sign should not be equated directly with the effective local charge experienced by ions inside the membrane during transport in 0.001–1 M HCl [52,53]. The zeta-potential data are used as evidence for proton-responsive interfacial chemistry.

Finally, the ordered composite architecture remains stable after aqueous exposure. After immersion in deionized water for 1 h and removal of excess surface water, the Ti_0.87_O_2_ (010) reflection shifts only from 9.41° to 9.36° (Figure 2I). This limited change is substantially smaller than the hydration-induced shifts reported for membranes assembled solely from two-dimensional nanosheets [21,54]. Additional XRD measurements (Figure S5), FTIR measurements (Figure S6), and mechanical data (Figure S7) further support the retention of the composite structure. The membrane therefore combines enhanced structural ordering and pH-responsive interfacial chemistry with the aqueous stability needed for transport measurements.

### 3.3. ANF Ordering, Proton Preference, and Cation Selectivity

**Figure 3:**
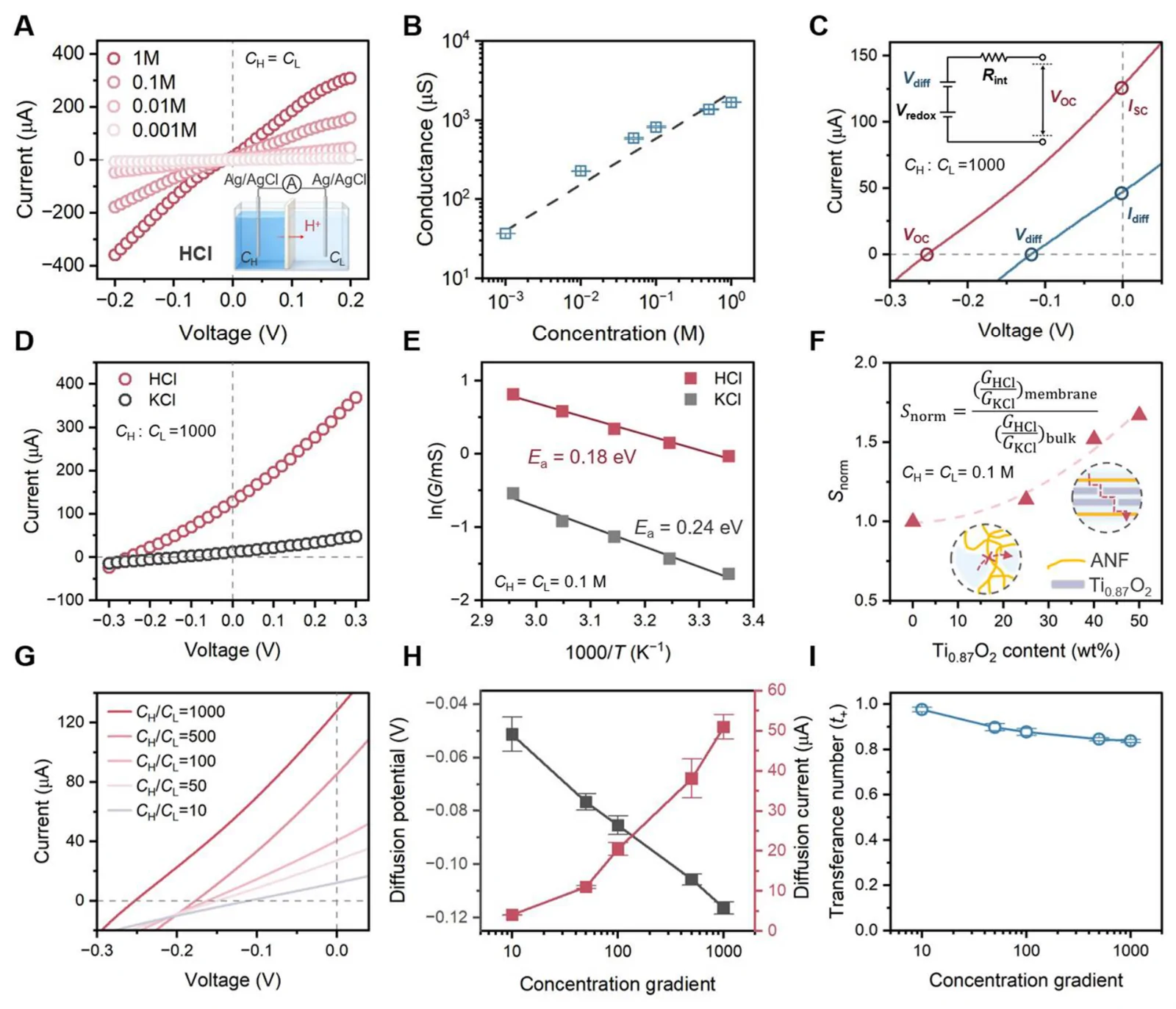
ANF ordering, proton preference, and cation selectivity. (**A**) *I*–*V* curves under symmetric HCl conditions. The inset shows the two-compartment cell used for transmembrane *I*–*V* measurements, with the membrane separating the HCl solutions and an Ag/AgCl electrode directly immersed in each compartment. (**B**) The corresponding membrane conductance as a function of HCl concentration. (**C**) *I*–*V* response under a 1000-fold HCl gradient measured with directly immersed and salt-bridge-isolated Ag/AgCl electrodes. (**D**) Comparison of HCl and KCl transport under identical 1000-fold concentration gradients. (**E**) Arrhenius plots for conductance under symmetric 0.1 M HCl and KCl conditions. (**F**) Normalized proton-preference factor as a function of Ti_0.87_O_2_ content. The schematic insets illustrate the proposed proton-transport pathways in the disordered ANF network (left) and the more ordered ANF/Ti_0.87_O_2_ architecture (right). Yellow lines and purple blocks represent ANFs and Ti_0.87_O_2_ nanosheets, respectively, while red dashed arrows indicate the proposed proton-transport pathways. (**G**) *I*–*V* curves under different HCl concentration gradients at *C*_L_ = 0.001 M. (**H**) Diffusion potential and diffusion current and (**I**) cation transference number as functions of the HCl concentration gradient.

Under symmetric HCl conditions (*C*_H_ = *C*_L_), the *I*–*V* curves recorded from 0.001 to 1 M HCl are approximately linear and pass through the origin (Figure 3A), indicating Ohmic transmembrane transport without appreciable built-in potential or ionic current rectification. The membrane conductance, *G*, obtained from the slope, increases from 36.8 μS at 0.001 M to 1657.0 μS at 1 M (Figure 3B).

A 1000-fold HCl gradient (1 M/0.001 M) displaced the *I*–*V* curve from the origin, giving a raw open circuit voltage (*V*_OC_) of 252 mV and a short circuit current (*I*_SC_) of 128 μA (Figure 3C). Forward and reverse scans (Figure S8) gave comparable responses, consistent with similar transport properties for the two membrane faces. Because directly immersed Ag/AgCl electrodes introduce a redox contribution that depends on chloride activity [53,55,56,57], electrodes isolated by a salt bridge were used to obtain the corrected *V*_diff_ (Section 2.5). The corrected measurement gave a *V*_diff_ magnitude of 116 mV and an *I*_diff_ of 47.4 μA. The polarity indicates preferential H^+^ diffusion from the high concentration side to the low concentration side, establishing preferential cation transport.

To compare HCl transport with the migration of a conventional hydrated cation, *I*–*V* curves were recorded under identical 1000-fold HCl and KCl gradients (Figure 3D). Across the scanned bias range, the HCl current is substantially higher. At zero applied bias, the HCl and KCl currents are 128 and 22.8 μA, respectively, corresponding to a 5.61-fold difference. This comparison establishes faster HCl transport than KCl under the same gradient, but it does not by itself isolate a membrane-specific proton contribution because H^+^ is intrinsically more mobile than K^+^ in bulk water [58,59].

Temperature-dependent measurements under symmetric 0.1 M HCl and KCl conditions (Figure S9) provide an independent kinetic comparison. HCl exhibits the higher conductance throughout the measured temperature range, and ln *G* varies approximately linearly with 1000/*T* (Figure 3E). Arrhenius fitting gives apparent activation energies (*E*_a_) of 0.18 eV for HCl transport and 0.24 eV for KCl transport. The lower HCl barrier supports a kinetic distinction between the two electrolytes, although the macroscopic *E*_a_ does not by itself identify a specific microscopic hopping mechanism.

To remove the intrinsic bulk HCl/KCl mobility difference, we define a dimensionless normalized proton-preference factor:

$$S_{norm}=\frac{{\left(G_{HCl}/G_{KCl}\right)}_{membrane}}{{\left(G_{HCl}/G_{KCl}\right)}_{bulk}}$$

Here, *G* is the conductance measured at fixed geometry, and values of *S*_norm_ above unity indicate that the membrane enhances the HCl/KCl conductance ratio relative to that in the bulk solutions. The underlying LSV curves for the bulk solution, the ANF membrane, and the composition series are provided in Figure S10. The ANF membrane gives *S*_norm_ ≈ 1.0 (Figure 3F). The composition-dependent structural orientation and ion-transport parameters are summarized in Table 1. With increasing Ti_0.87_O_2_ content, the azimuthal FWHM of the composite membranes decreased from 62.5° to 35.5°, while the HCl conductance increased more strongly than the KCl conductance, resulting in a progressive increase in *S*_norm_ from 1.00 to 1.67. The parallel increase in ANF ordering and *S*_norm_, together with the lower apparent activation energy for HCl, is consistent with a possible contribution of nanosheet-associated ordering to the formation of a more favorable hydrogen-bonded interfacial environment involving amide groups, surface hydroxyls, and water. At such interfaces, changes in local protonation and confinement can alter the organization and dynamics of interfacial water, thereby modifying the hydrogen-bonding environment available for proton transport. Such an environment may favor proton transport relative to conventional hydrated cations. The insets in Figure 3F schematically illustrate the proposed relationship between ANF organization and proton transport. Additional LSV curves under 1000-fold concentration gradients of HCl, KCl, NaCl, LiCl, CaCl_2_, and MgCl_2_ are provided in Figure S11.

The response to the driving force was evaluated by fixing the low-concentration side at *C*_L_ = 0.001 M and increasing *C*_H_/*C*_L_ to 10, 50, 100, 500, and 1000 (Figure 3G). As the gradient increases, the voltage intercept shifts progressively and the zero-bias diffusion current rises. After removal of the Ag/AgCl electrode contribution, the magnitude of *V*_diff_ increases from 56 mV at a 10-fold gradient to 116 mV at a 1000-fold gradient, while the diffusion current in this gradient series increases from 4.08 to 47.4 μA (Figure 3H). Thus, a larger proton chemical-potential difference increases both the electromotive force and the net transmembrane flux.

The cation transference number, *t*_+_, was obtained from the corrected diffusion potential using the Planck–Henderson relation described in Section 2.5 [40,41,42]. In HCl, *t*_+_ predominantly represents the H^+^ contribution. It decreases from 0.98 at a 10-fold gradient to 0.84 at a 1000-fold gradient (Figure 3I), potentially reflecting stronger electrostatic screening, concentration polarization, and reduced effective selectivity at high ionic strength [60,61]. Nevertheless, the membrane retains pronounced cation selectivity across the investigated gradient range. Collectively, these measurements establish cation selectivity under HCl gradients and a proton preference relative to K^+^ in the same membrane in which ANF ordering is enhanced.

### 3.4. RED-Type Proton Gradient Energy Conversion and Device Integration

Electrical output in the RED-type configuration was evaluated across HCl concentration gradients under external loads, using Ag/AgCl electrodes isolated by salt bridges (Section 2.6).

**Figure 4:**
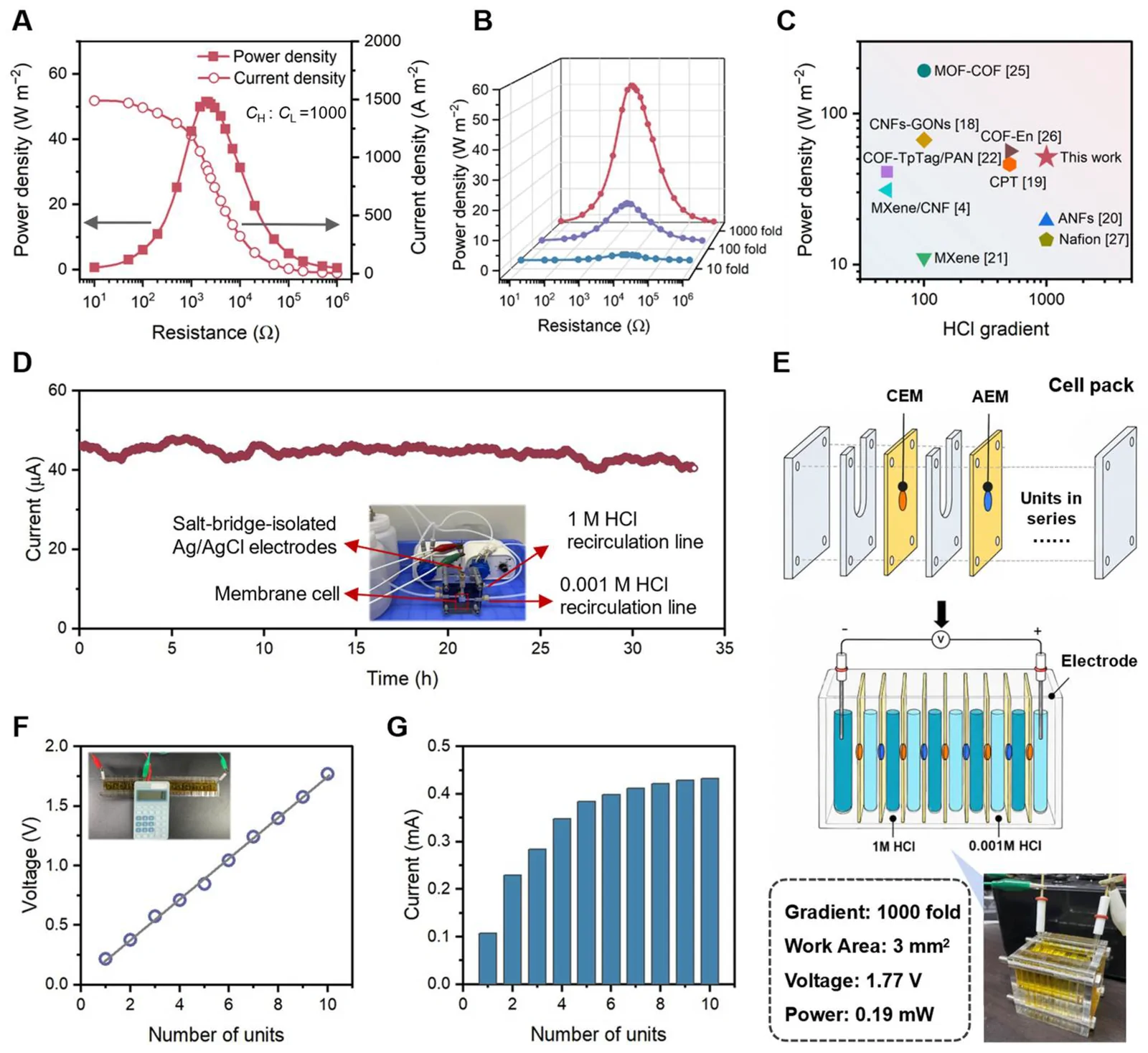
RED-type proton gradient energy conversion and device integration. (**A**) Current density and power density as functions of external load resistance under a 1000-fold HCl gradient; filled symbols represent power density, whereas open symbols represent current density. (**B**) Load-dependent power density under 10-, 100-, and 1000-fold HCl gradients. (**C**) Comparison of the maximum power density with literature-reported ion-selective membranes. Reference numbers are indicated alongside the corresponding systems, and detailed testing conditions are summarized in Table S2. (**D**) Short-circuit-current stability under a 1000-fold HCl gradient (1 M/0.001 M HCl). The inset shows the recirculating test setup, including the membrane cell, salt-bridge-isolated Ag/AgCl electrodes, and the 1 M and 0.001 M HCl recirculation lines. (**E**) Architecture and output of the integrated RED-type proton gradient device; CEM and AEM denote cation-exchange membrane and anion-exchange membrane, respectively. The ellipsis indicates additional repeated units connected in series. (**F**) Open-circuit voltage and (**G**) short-circuit current as functions of the number of integrated units.

Under a 1 M/0.001 M HCl gradient, increasing *R*_L_ from approximately 10^1^ to 10^6^ Ω decreases the current density from approximately 1490 A m^−2^ to nearly zero (Figure 4A). The power density consequently displays a single maximum, reaching an average maximum power density of 51.52 W m^−2^ at approximately 2000 Ω. The power density was directly calculated from the measured load current at each resistance rather than estimated from the open-circuit voltage and short-circuit current. The maximum reflects impedance matching between the external load and the internal resistance of the membrane cell. Load scans at 10-, 100-, and 1000-fold HCl gradients yield maximum power densities of approximately 1.88, 13.40, and 51.52 W m^−2^, respectively (Figure 4B). This gradient dependence tracks the increases in diffusion potential and diffusion current in Figure 3, directly connecting the proton chemical-potential difference to usable electrical output.

Figure 4C compares the maximum power density of the ANF/Ti_0.87_O_2_ membrane under a 1000-fold HCl gradient with literature values for ion-selective membranes tested under their reported conditions. The maximum of 51.52 W m^−2^ represents a high membrane area-normalized output among reported proton gradient membrane systems under the present microscale test conditions. Because electrolyte concentrations and effective areas differ among reports, the comparison should be interpreted in the context of the conditions listed in Table S2.

Continuous short-circuit operation was evaluated under the 1000-fold HCl gradient (Figure 4D). The current remains mainly within 40–48 μA over approximately 33 h, decreasing from 45.9 μA initially to 40.43 μA at the end of the test. The corresponding current retention is 88.1%. Although a gradual decline becomes evident at longer times, the membrane sustains continuous proton gradient-driven current throughout the test, supporting the operational stability of the membrane under these conditions.

For modular integration, alternating ANF/Ti_0.87_O_2_ and PiperION A20 membranes formed a device containing 10 units, with 1 M and 0.001 M HCl supplied to adjacent channels (Figure 4E). With an effective area of 3 mm^2^, the stack generated an open circuit voltage of 1.77 V and a short circuit current of approximately 0.43 mA, corresponding to a maximum output power of 0.19 mW, and it directly powered a low power calculator.

The open-circuit voltage increases approximately linearly with unit number, from approximately 0.215 V for one unit to 1.77 V for 10 units, corresponding to an incremental contribution of approximately 0.17–0.18 V per added unit (Figure 4F). This voltage addition confirms consistent polarity across the stack. The short-circuit current rises from approximately 0.11 mA for one unit to approximately 0.43 mA for 10 units (Figure 4G). In contrast to the nearly linear voltage scaling, the current increases rapidly over the first several units and approaches a plateau after approximately six units, consistent with growing limitations from stack resistance and mass-transfer polarization. Thus, the multi-unit measurements demonstrate consistent voltage addition and modular integration of the proton gradient membrane system. It should be noted that the high area-normalized power density obtained in the present microscale membrane cell does not directly represent large-scale power-generation capability. In practical RED systems, increasing membrane area and unit number can introduce additional stack resistance, mass-transfer limitations, and hydraulic losses, making total and net power outputs important metrics for scale-up. Therefore, the present 10-unit device demonstrates modular voltage integration, while further evaluation at larger membrane areas and stack sizes will be required to assess practical scalability.

## 4. Conclusions

Freestanding ANF/Ti_0.87_O_2_ membranes approximately 3 μm thick were prepared by TfOH co-dispersion, spin coating, aqueous regeneration, and confined drying. Ti_0.87_O_2_ incorporation is associated with changes in the local amide interaction environment, substantially enhanced ANF packing, and preferential orientation, while the compact composite architecture remains stable after aqueous exposure. The transport data provide three distinct levels of evidence: diffusion potentials and *t*_+_ establish cation selectivity under HCl gradients; bulk-normalized *S*_norm_ and the lower apparent activation energy for HCl support proton preference relative to K^+^ beyond the bulk mobility difference. *S*_norm_ increases from approximately 1.0 for the ANF membrane to 1.67 at a Ti_0.87_O_2_ mass fraction of 0.5. Under a 1000-fold HCl gradient, the membrane reaches 51.52 W m^−2^ and retains 88.1% of its initial short-circuit current after approximately 33 h. Alternating the membrane with an anion-exchange membrane yields a 10-unit device with an output of 1.77 V. The distinct contribution of this study is therefore the correlation between Ti_0.87_O_2_-associated ANF organization and proton preference within an ultrathin regenerated architecture. More broadly, regulating rigid polymer organization at two-dimensional interfaces may provide a useful strategy for designing proton-selective membranes, while future studies should clarify how interfacial ordering and hydration jointly govern proton transport and whether this principle can be extended to other rigid polymer systems.

## Figures and Tables

**Table 1:** Summary of the structural orientation and ion-transport properties of ANF/Ti_0.87_O_2_ membranes with different Ti_0.87_O_2_ contents.

Ti_0.87_O_2_ Content (wt%)	Azimuthal FWHM (°)	HCl Ionic Conductance (mS)	KCl Ionic Conductance (mS)	Proton-Selectivity Parameter, *S*_norm_
0	n.d. *	0.288	0.096	1.00
25	62.5	0.338	0.099	1.14
40	46.7	0.605	0.133	1.52
50	35.5	0.971	0.194	1.67

* n.d., not determined because no resolvable azimuthal-orientation peak was observed.

## Data Availability

The original contributions presented in this study are included in the article/Supplementary Material. Further inquiries can be directed to the corresponding author.

## Supplementary Materials

The following supporting information can be downloaded at https://www.mdpi.com/article/10.3390/nano16181149/s1: Figure S1: Preparation of the ANF/Ti_0.87_O_2_ membrane. (A) ANF/Ti_0.87_O_2_ dispersion in TfOH. (B) Confinement module used for vacuum drying of the ANF/Ti_0.87_O_2_ membrane. Figure S2: Morphology and size statistics of exfoliated Ti_0.87_O_2_ nanosheets and cross-sectional elemental distribution of the composite membrane. (A) AFM image. (B) Thickness distribution. (C) Lateral-size distribution. (D) Cross-sectional SEM image of the ANF/Ti0.87O2 membrane and corresponding EDS elemental maps of (E) N and (F) Ti. Figure S3: XPS analysis of the constituent materials and membrane. (A) Survey spectrum of Ti_0.87_O_2_. (B) Survey spectrum of ANF. (C) Ti 2p spectra of Ti_0.87_O_2_ and the ANF/Ti_0.87_O_2_ membrane. (D) N 1s spectra of ANF and the ANF/Ti_0.87_O_2_ membrane. Figure S4: FTIR spectra of the ANF membrane and ANF/Ti_0.87_O_2_ membrane. (A) Full FTIR spectra of ANF and ANF/Ti_0.87_O_2_ membranes. (B) Expanded N–H stretching region for three independently prepared ANF membranes and three independently prepared ANF/Ti_0.87_O_2_ membranes. (C) Expanded amide I region for three independently prepared ANF membranes and three independently prepared ANF/Ti_0.87_O_2_ membranes. (D) Average peak positions of the N–H stretching band for ANF and ANF/Ti_0.87_O_2_ membranes. (E) Average peak positions of the amide I band for ANF and ANF/Ti_0.87_O_2_ membranes. Error bars represent standard deviation from three independently prepared membranes. Figure S5: XRD patterns of the ANF/Ti_0.87_O_2_ membrane in dry and wet states. Figure S6: FTIR spectra of the ANF/Ti_0.87_O_2_ membrane in dry and wet states. Figure S7: Tensile stress–strain curve of the ANF/Ti_0.87_O_2_ membrane. Figure S8: LSV curves of the ANF/Ti_0.87_O_2_ membrane measured with forward and reverse orientations of a 1000-fold HCl concentration gradient. Figure S9: Temperature-dependent LSV curves of the ANF/Ti_0.87_O_2_ membrane in symmetric electrolyte solutions. (A) 0.1 M HCl. (B) 0.1 M KCl. Figure S10: LSV curves of bulk solution and membranes with different Ti_0.87_O_2_: ANF mass ratios in symmetric 0.1 M HCl and 0.1 M KCl. (A) Bulk solution. (B) ANF membrane. (C) Ti_0.87_O_2_: ANF = 1:3. (D) Ti_0.87_O_2_: ANF = 2:3. Figure S11: LSV curves of the ANF/Ti_0.87_O_2_ membrane under 1000-fold concentration gradients of HCl, KCl, NaCl, LiCl, CaCl_2_, and MgCl_2_. Table S1: Calculation of cation transference numbers for the ANF/Ti_0.87_O_2_ membrane under different HCl concentration gradients. Table S2: Comparison of reported proton gradient osmotic power-generation membranes. References [62,63,64] are cited in the supplementary materials.
