# Supplementary material for: Ti0.87O2 Nanosheet-Associated Aramid Nanofiber Ordering in Ultrathin Membranes for Proton Gradient Energy Conversion
Source: Nanomaterials (Basel). 2026 Sep 14;16(18):1149. doi: 10.3390/nano16181149 (PMC13610094; doi:10.3390/nano16181149)
Supplement: Supplementary file 1 [file nanomaterials-16-01149-s001.zip › nanomaterials-4548428-supplementary.pdf]

$Ti_{0.87}O_2$ Nanosheet-Associated Aramid Nanofiber Ordering in Ultrathin Membranes for Proton Gradient Energy Conversion

Supporting Information

1. Experimental Section

Materials. Kevlar chopped fibers (DuPont; Kevlar®, K29), trifluoromethanesulfonic acid (TfOH; Aladdin; ≥99.5%), TiO_2 (Aladdin; ≥99.9%), K_2CO_3 (Aladdin; ≥99%), Li_2CO_3 (Aladdin; ≥99.99%), tetra-n-butylammonium hydroxide (TBAOH; Aladdin; ～25% in H2O), hydrochloric acid (HCl; Guangzhou Chemical Reagent Factory; 36–38%), potassium chloride (KCl; Aladdin; ≥99.8%), sodium chloride (NaCl; Aladdin; ≥99.8%), lithium chloride (LiCl; Aladdin; ≥98%), calcium chloride ($CaCl_2$; Aladdin; ≥97%), magnesium chloride ($MgCl_2$; Aladdin; ≥98%), and PiperION® A20 anion-exchange membranes (Versogen) were used. Deionized water (18.2 MΩ·cm) was used for membrane preparation and electrolyte solutions.

Preparation of $Ti_{0.87}O_2$ nanosheets. $Ti_{0.87}O_2$ nanosheets were prepared by soft-chemical exfoliation of a layered potassium lithium titanate precursor following previously reported methods. TiO_2, K_2CO_3, and Li_2CO_3 were mixed at a molar ratio of 1.73:0.40:0.14 and calcined at 900 °C for 20 h to obtain layered $K_{0.8}Ti_{1.73}Li_{0.27}O_4$. The resulting precursor was stirred in 0.5 M HCl at room temperature for 48 h to produce protonated $H_{1.07}Ti_{1.73}O_4 \cdot H_2O$, which was collected by filtration, thoroughly washed with deionized water, and air-dried at room temperature. The protonated titanate was subsequently treated with an aqueous tetra-*n*-butylammonium hydroxide (TBAOH) solution at a TBA^+/H^+ molar ratio of 1:1 and shaken for 7 days to obtain a stable $Ti_{0.87}O_2$ nanosheet suspension. The suspension was freeze-dried to obtain $Ti_{0.87}O_2$ solids.

Preparation of ANF/$Ti_{0.87}O_2$ membranes. Kevlar chopped fibers were dispersed in TfOH at an ANF concentration of 15 mg mL^{-1}. $Ti_{0.87}O_2$ solids were added at the designed mass ratio and dispersed to obtain a homogeneous ANF/$Ti_{0.87}O_2$ dispersion. Unless otherwise specified, the ANF:$Ti_{0.87}O_2$ mass ratio was 1:1. For the composition-dependent samples, the $Ti_{0.87}O_2$:ANF mass ratios were 1:3 and 2:3. The dispersion was spin-coated onto a silicon substrate at 1000 rpm for 20 s. The coated film was immersed in deionized water for 1 h to exchange the solvent and remove TfOH. The hydrated membrane was then vacuum-dried under confinement at 40 °C for 24 h. The confinement setup is shown in Figure S1B. A freestanding ANF/$Ti_{0.87}O_2$ membrane was obtained after drying.

Preparation of ANF control membranes. ANF control membranes were prepared from the 15 mg mL^{-1} ANF/TfOH dispersion without $Ti_{0.87}O_2$ solids. The spin-coating speed was 1000 rpm. The spin-coating time, water-immersion procedure, and confined-vacuum-drying conditions were the same as those used for the ANF/$Ti_{0.87}O_2$ membranes.

2. Characterization

Scanning electron microscopy (SEM). SEM images of the exfoliated $Ti_{0.87}O_2$ nanosheets and membrane cross sections were acquired using a Hitachi SU8010 scanning electron microscope at an accelerating voltage of 10 kV.

Atomic force microscopy (AFM). AFM images of the exfoliated $Ti_{0.87}O_2$ nanosheets were acquired using a Bruker Dimension Icon system in tapping mode.

X-ray photoelectron spectroscopy (XPS). XPS spectra of $Ti_{0.87}O_2$, ANF, and the ANF/$Ti_{0.87}O_2$ membrane were recorded using a Thermo Scientific K-Alpha system with Al Kα radiation (hv = 1486.6 eV) and a beam spot of 400 μm. Survey spectra and high-resolution Ti 2p and N 1s spectra were collected. Survey scans were recorded with a pass energy of 100 eV and a step size of 1 eV; high-resolution scans were recorded with a pass energy of 50 eV and a step size of 0.1 eV, with at least five cycles accumulated for each narrow scan. The base pressure in the analysis chamber was better than 5.0×10^{-7} mbar. The C 1s spectrum was fitted first, and the binding-energy scale was charge-corrected by referencing the C–C/C=C component to 284.8 eV. The same binding-energy correction was then applied to all high-resolution spectra of the corresponding sample.

Fourier-transform infrared spectroscopy (FTIR). FTIR spectra of the ANF membrane and the ANF/$Ti_{0.87}O_2$ membrane were collected using a PerkinElmer Spectrum 3 FTIR spectrometer equipped with a universal ATR accessory over the range 4000–400 cm^{-1}, with a spectral resolution of 1 cm^{-1} and 16 scans. Dry and wet ANF/$Ti_{0.87}O_2$ membranes were also compared.

Powder X-ray diffraction (PXRD). XRD patterns of $Ti_{0.87}O_2$, the ANF membrane, and the ANF/$Ti_{0.87}O_2$ membrane were recorded using a Bruker D8 ADVANCE diffractometer with Cu Kα radiation (λ = 1.5418 Å) at 40 kV and 15 mA. Data were recorded over 2θ = 5–40° with a step size of 0.01°. For wet-state XRD, the ANF/$Ti_{0.87}O_2$ membrane was immersed in deionized water for 1 h, and excess surface water was removed before measurement.

Two-dimensional wide-angle X-ray scattering (2D WAXS). Two-dimensional WAXS measurements of the ANF and ANF/$Ti_{0.87}O_2$ membranes were performed using a Xeuss 2.0 system (Xenocs, France) equipped with a Cu Kα source operating at a tube power of 30 W (λ = 1.54189 Å) and a Pilatus 3R 300K detector with a pixel size of 172 μm. The X-ray beam was incident perpendicular to the membrane plane. The sample-to-detector distance was 82 mm, and the total exposure time was 600 s. The two-dimensional scattering patterns were radially integrated to

obtain one-dimensional intensity profiles as a function of the scattering vector q, defined as $q = 4\pi \sin \theta/\lambda$. Azimuthal intensity profiles were extracted from the relevant reflections to evaluate the preferred orientation of the membranes.

Zeta-potential measurements. The pH-dependent zeta potential of the ANF/$Ti_{0.87}O_2$ membrane surface was measured using a SurPASS 3 electrokinetic analyzer (Anton Paar, Austria) in streaming-potential mode. This measurement probed the membrane surface; it was not an electrophoretic measurement of a $Ti_{0.87}O_2$ particle dispersion.

Mechanical testing. Uniaxial tensile tests of the ANF/$Ti_{0.87}O_2$ membrane were performed using an Instron 5943 universal testing system.

Ion-transport measurements

Symmetric electrolyte measurements. ANF/$Ti_{0.87}O_2$ membranes were mounted between two chambers of a custom two-compartment electrochemical cell, with an effective exposed membrane area of 0.03 mm^2. Homemade Ag/AgCl electrodes were used for direct-contact measurements, whereas commercial single-salt-bridge Ag/AgCl electrodes (Gaoss Union) were used when the electrode redox contribution had to be minimized. A Bio-Logic SP-200 electrochemical workstation was used to apply the transmembrane voltage and record the current. For symmetric measurements, both chambers contained electrolyte solutions of the same concentration. HCl concentrations of 0.001, 0.01, 0.1, and 1 M were tested, and symmetric 0.1 M HCl and 0.1 M KCl solutions were used for comparative measurements. The voltage was scanned from −0.3 to 0.3 V at 10 mV s^{-1} after an equilibration time of 5 s. The transmembrane conductance, G, was obtained from the slope of the linear region of each LSV (I–V) curve.

Temperature-dependent ion transport. Temperature-dependent LSV measurements shown in Figure S9 were performed in symmetric 0.1 M HCl and symmetric 0.1 M KCl at 25, 35, 45, 55, and 65 °C using a thermostatic water bath. At each temperature, the electrochemical cell was equilibrated for 30 min before measurement. Conductance was obtained from the slope of the linear region, and the apparent activation energy was determined by fitting ln G versus 1000/T according to the Arrhenius relationship

$$\ln G = \ln G_0 - E_a/RT$$

where G_0 is the pre-exponential factor, E_a is the apparent activation energy, R is the gas constant, and T is the absolute temperature.

$$S_{norm} = \frac{(G_{HCl}/G_{KCl})_{membrane}}{(G_{HCl}/G_{KCl})_{bulk}}$$

Bulk-normalized HCl/KCl transport ratio. The normalized factor was calculated as

where $S_{\mathrm{norm}} = 1$ indicates that the HCl/KCl transport ratio in the membrane is identical to that in the bulk solution, whereas $S_{\mathrm{norm}} > 1$ indicates membrane-associated HCl preference relative to KCl beyond the bulk conductivity ratio. This metric does not by itself establish a specific microscopic proton-transfer mechanism.

Concentration-gradient measurements. The membrane was mounted between high-concentration (C_H) and low-concentration (C_L) electrolyte reservoirs. For the HCl-gradient series, C_L was fixed at 0.001 M and C_H/C_L was set to 10, 50, 100, 500, or 1000. HCl and KCl were compared under 1000-fold concentration gradients. Additional 1000-fold gradient measurements were performed with HCl, KCl, NaCl, LiCl, $CaCl_2$, and $MgCl_2$ as shown in Figure S11; the exact high- and low-concentration values for these additional electrolytes were 1 M and 0.001 M, respectively.

Correction of electrode redox potentials. In measurements made with Ag/AgCl electrodes directly contacting the two electrolyte solutions, the raw open-circuit voltage, V_{OC}, contained contributions from both the membrane diffusion potential, V_{diff}, and the difference in electrode redox potentials, V_{redox}, arising from the different chloride activities. Salt-bridge Ag/AgCl electrodes were therefore used to minimize the electrode contribution and obtain the corrected membrane diffusion potential. V_{diff} and the diffusion current, I_{diff}, were obtained from the voltage-axis and current-axis intercepts, respectively, of the corrected I-V curve.

Cation transference number. The cation transference number, t_+, was calculated from the corrected diffusion potential using the Planck–Henderson diffusion-potential relationship

$$t_+ = 1/2\ [1 + zF|V_{diff}|/(RT\ \ln(a_H/a_L))]$$

where $a_H = \gamma_H\ C_H$ and $a_L = \gamma_L\ C_L$; V_{diff} is the corrected diffusion potential; γ is the activity coefficient [43]; C is the electrolyte concentration; R is the gas constant; T is the absolute temperature; F is the Faraday constant; and z = 1 for HCl. The activity coefficients and calculated values are listed in Table S1 without modification.

Osmotic power generation

Single-membrane osmotic power measurements. An ANF/$Ti_{0.87}O_2$ membrane was placed between high- and low-concentration HCl solutions and connected to salt-bridge-isolated Ag/AgCl electrodes. An external variable load resistance, R_L, was connected to the circuit, and the load current, *I*, was recorded at each resistance. Load-resistance measurements were performed under 10-, 100-, and 1000-fold HCl concentration gradients with C_L = 0.001 M. The power density, *P*, was calculated as

$$P = I^2 R_L / A$$

where I is the load current, R_L is the external load resistance, and A is the effective membrane area (0.03 mm^2 for the single-membrane cell).

Stability test. The short-circuit current was continuously monitored for approximately 33 h under a 1000-fold HCl concentration gradient (1 M/0.001 M).

Integrated osmotic power-generation device. The integrated device was assembled from end plates, flow-channel plates, sealing components, ANF/$Ti_{0.87}O_2$ membranes, and PiperION® A20 anion-exchange membranes. The two membrane types were arranged alternately, and 1 M and 0.001 M HCl were introduced into adjacent channels. The effective working area was 3 mm^2. Devices containing up to 10 integrated units were tested. Open-circuit voltage and short-circuit current were recorded as functions of the number of integrated units.

3. Results

A B

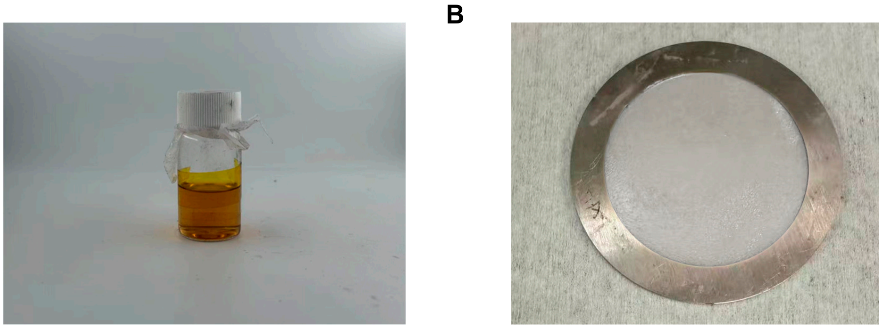

Figure S1. Preparation of the ANF/$Ti_{0.87}O_2$ membrane. (A) ANF/$Ti_{0.87}O_2$ dispersion in TfOH. (B) Confinement module used for vacuum drying of the ANF/$Ti_{0.87}O_2$ membrane.

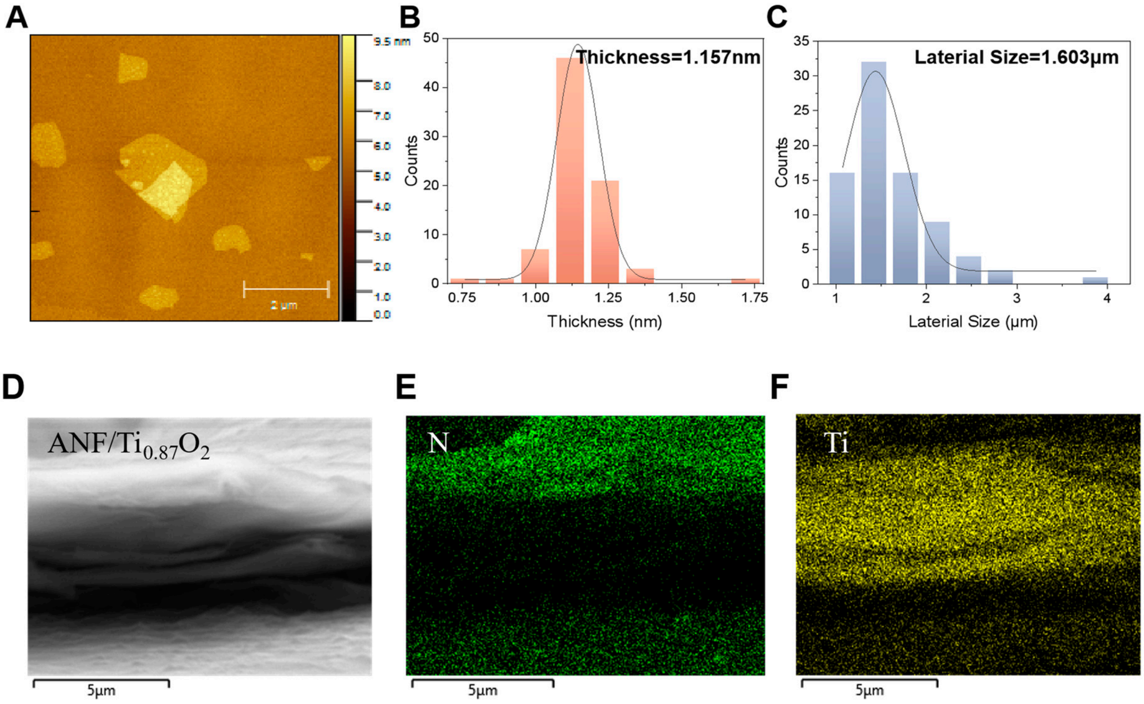

Figure S2. Morphology and size statistics of exfoliated $Ti_{0.87}O_2$ nanosheets and cross-sectional elemental distribution of the composite membrane. (A) AFM image. (B) Thickness distribution. (C) Lateral-size distribution. (D) Cross-sectional SEM image of the ANF/$Ti_{0.87}O_2$ membrane and corresponding EDS elemental maps of (E) N and (F) Ti.

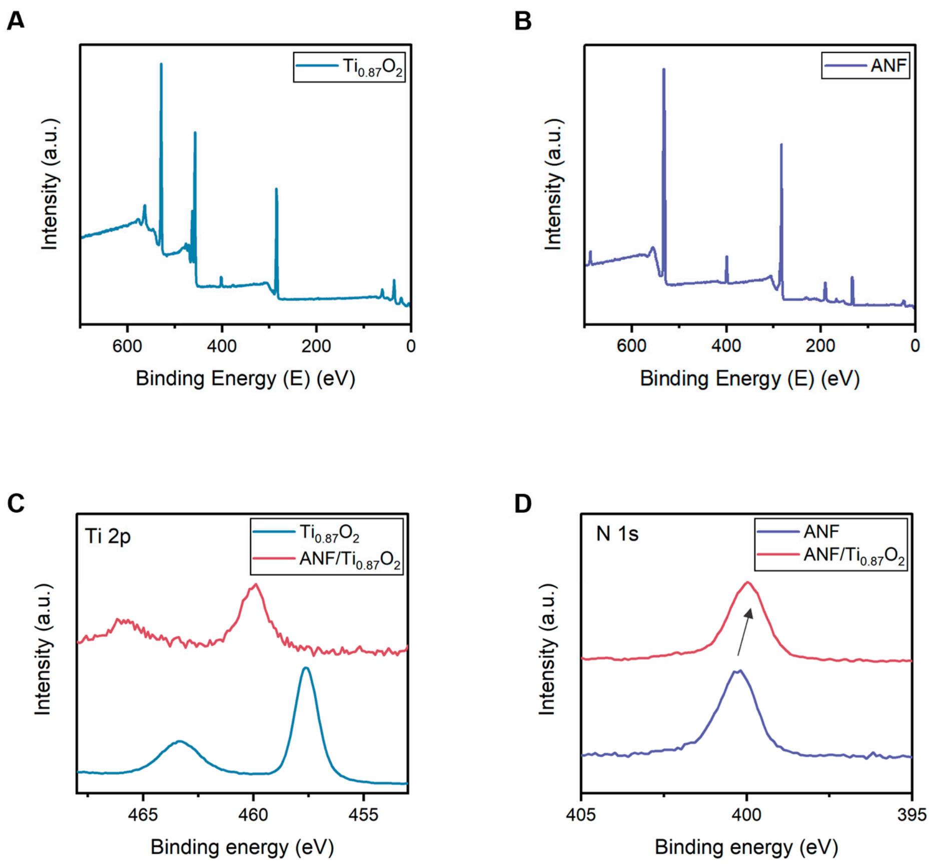

Figure S3. XPS analysis of the constituent materials and membrane. (A) Survey spectrum of $Ti_{0.87}O_2$. (B) Survey spectrum of ANF. (C) Ti 2p spectra of $Ti_{0.87}O_2$ and the ANF/$Ti_{0.87}O_2$ membrane. (D) N 1s spectra of ANF and the ANF/$Ti_{0.87}O_2$ membrane.

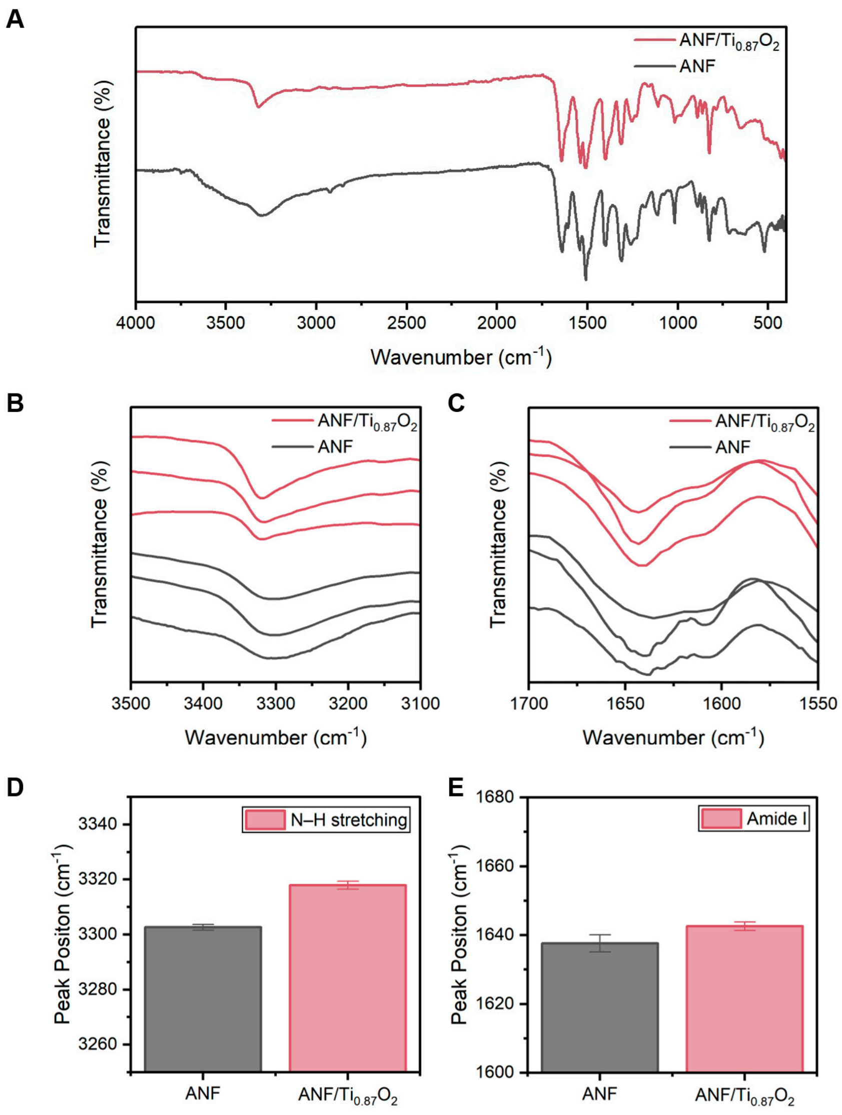

Figure S4. FTIR spectra of the ANF membrane and ANF/$Ti_{0.87}O_2$ membrane.

(A) Full FTIR spectra of ANF and ANF/$Ti_{0.87}O_2$ membranes. (B) Expanded N–H stretching region for three independently prepared ANF membranes and three independently prepared ANF/$Ti_{0.87}O_2$ membranes. (C) Expanded amide I region for three independently prepared ANF membranes and three independently prepared ANF/$Ti_{0.87}O_2$ membranes. (D) Average peak positions of the N–H stretching band for ANF and ANF/$Ti_{0.87}O_2$ membranes. (E) Average peak positions of the amide I band for ANF and ANF/$Ti_{0.87}O_2$ membranes. Error bars represent standard deviation from three independently prepared membranes.

To assess the reproducibility of the FTIR peak shifts, three separate membranes of each type were

prepared using the same procedure and measured. Figure S4A presents representative full-range spectra, while Figure S4B,C show the expanded N–H stretching and amide I regions for all three membranes of each type. The spectra are vertically offset for clarity, so their relative vertical positions do not represent differences in absolute transmittance. The N–H stretching peak positions are 3302.65 ± 1.07 cm^{-1} for ANF and 3317.91 ± 1.42 cm^{-1} for ANF/$Ti_{0.87}O_2$, while the amide I peak positions are 1637.63 ± 2.50 and 1642.60 ± 1.27 cm^{-1}, respectively (Figure S4D,E; mean ± standard deviation, n = 3 separately prepared membranes). For both bands, all three composite membranes exhibit higher peak wavenumbers than the ANF controls, supporting the reproducibility of the shift direction across membrane samples.

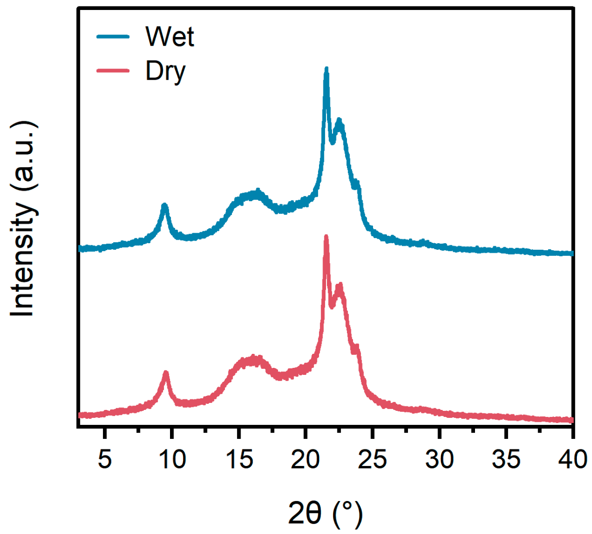

Figure S5. XRD patterns of the ANF/$Ti_{0.87}O_2$ membrane in dry and wet states.

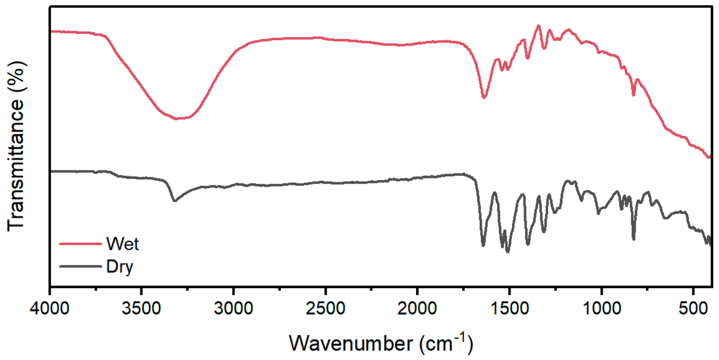

Figure S6. FTIR spectra of the ANF/$Ti_{0.87}O_2$ membrane in dry and wet states.

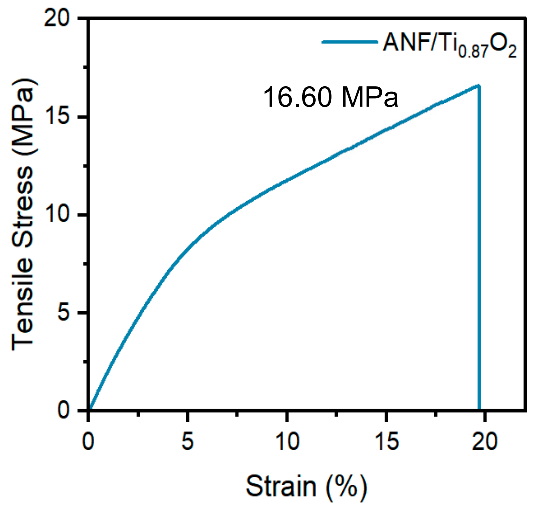

Figure S7. Tensile stress-strain curve of the ANF/$Ti_{0.87}O_2$ membrane.

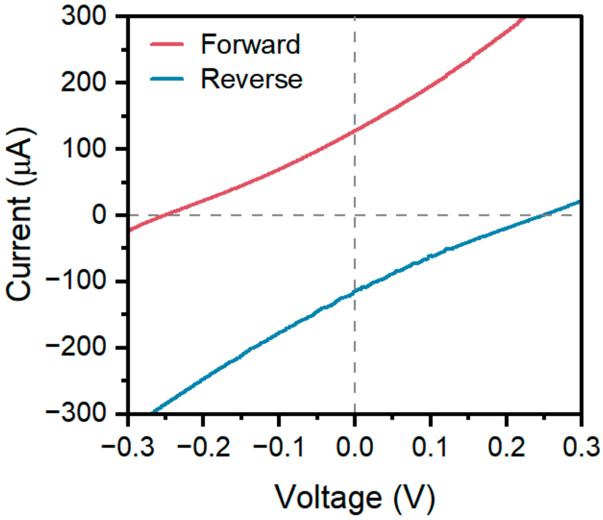

Figure S8. LSV curves of the ANF/$Ti_{0.87}O_2$ membrane measured with forward and reverse orientations of a 1000-fold HCl concentration gradient.

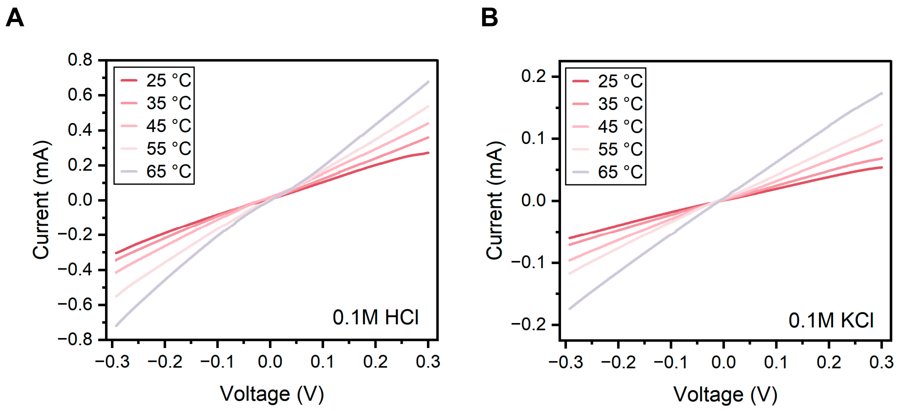

Figure S9. Temperature-dependent LSV curves of the ANF/$Ti_{0.87}O_2$ membrane in symmetric electrolyte solutions. (A) 0.1 M HCl. (B) 0.1 M KCl.

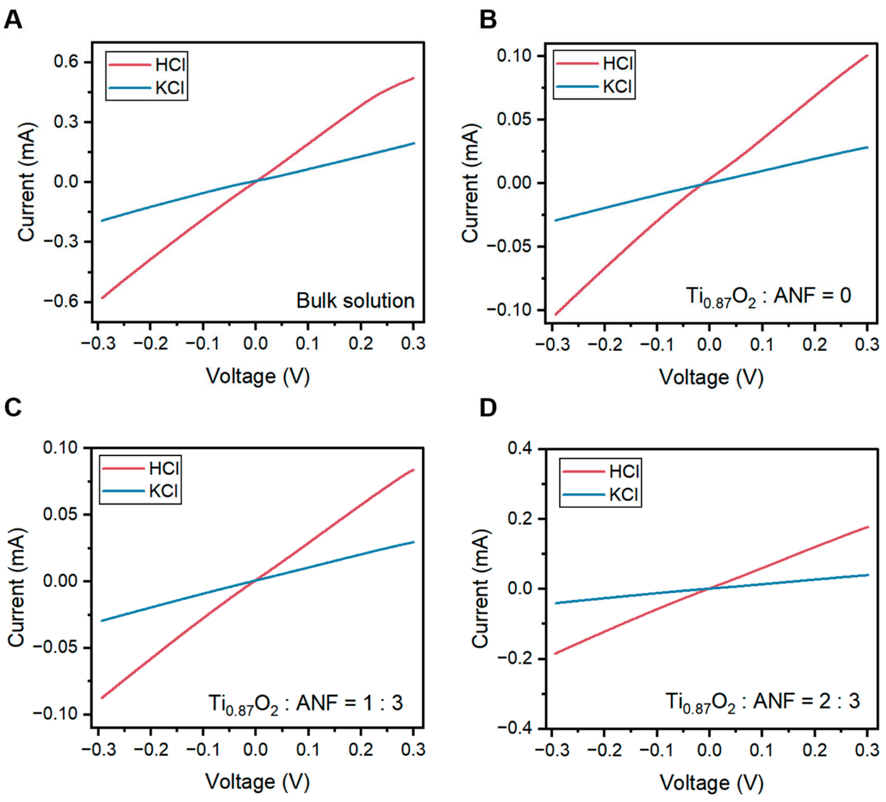

Figure S10. LSV curves of bulk solution and membranes with different $Ti_{0.87}O_2$: ANF mass ratios in symmetric 0.1 M HCl and 0.1 M KCl. (A) Bulk solution. (B) ANF membrane. (C) $Ti_{0.87}O_2$: ANF = 1:3. (D) $Ti_{0.87}O_2$: ANF = 2:3.

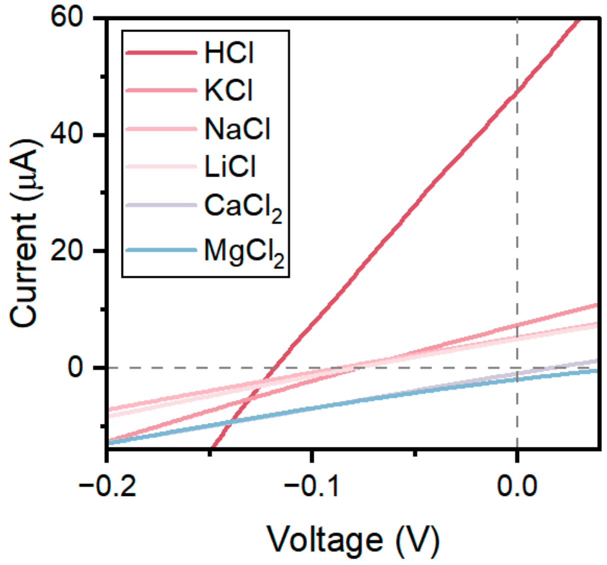

Figure S11. LSV curves of the ANF/$Ti_{0.87}O_2$ membrane under 1000-fold concentration gradients of HCl, KCl, NaCl, LiCl, $CaCl_2$, and $MgCl_2$.

Table S1. Calculation of cation transference numbers for the ANF/$Ti_{0.87}O_2$ membrane under different HCl concentration gradients.

C_H/C_L	Diffusion potential, V_{diff} (V)	γ_H	C_H (M)	γ_L	C_L (M)	z	t_+
1000	-0.116	0.811	1	0.965	0.001	1	0.837
500	-0.106	0.759	0.5	0.965	0.001	1	0.845
100	-0.085	0.797	0.1	0.965	0.001	1	0.878
50	-0.077	0.832	0.05	0.965	0.001	1	0.898
10	-0.055	0.905	0.01	0.965	0.001	1	0.977

* T = 298.15 K

Table S2. Comparison of reported proton-gradient osmotic power-generation membranes.

Membrane	Electrolyte concentration(mM)	Power density (W m^{-2})	Effective area (mm^2)	Reference
ANF/$Ti_{0.87}O_2$	1000/1 HCl	51.52	0.03	This work
MOF-COF	1000/10 HCl	192.9	0.03	[25]
	500/10 HCl	68.2		
ANFs	1000/1 HCl	19.5	0.03	[20]
	1000/1 HCl	1.02	3.14	
	1000/1 HCl	0.20	19.625	
	1000/1 HCl	0.16	78.5	
MXene	1000/10 (at 330K) HCl	11.1	0.03	[21]
	1000/10 HCl	6.5	0.03	
Nafion	1000/1 HCl	14.6	0.03	[27]
	1000/1 HCl	5.1	0.2	
	1000/1 HCl 333K	8.1	0.2	
	1000/1 HCl	0.85	12.5	
	5000/1000 HCl	2.13	12.5	
COF-TpTag/PAN	500/10 HCl	41.1	0.03	[22]
	500/10 H_2SO_4	97.1	0.03	
	500/10 H_3PO_4	6.8	0.03	
TpPa-TMC/PES	2000/40 H_2SO_4	135.65	0.03	[23]
SPX membrane	500 HCl/500 NaCl	0.44	0.2	[28]
	500 HCl/500 LiCl	0.55	0.2	
CNFs-GONs	500/10 NaCl	5.26	0.03	[18]

	500/5 HCl	67.08	0.03	
TpPa–SO_3H COF	5000/500 HCl	55	7	[29]
bamboo-mimic $Ti_3C_2T_x$	500 HCl/10 NaOH	163.87	0.03	[62]
	500 HCl/10 NaOH	30.52	0.196	
	500 HCl/10 HCl	7.28	0.196	
MXLDH-86	1000 HCl/1000 NaOH	8.75	0.03	[63]
MXene/CNF	500 HCl /10 NaOH	80.32	0.03	[4]
	500 HCl /10 HCl	31.17	0.03	
AAM/BC	500 HCl/10 KOH	45.5	0.03	[64]
COF-En	500 /10 H_2SO_4	99.3	0.03	[26]
	500 /10 HCl	56.6		
	1000 /10 H_2SO_4	243.1		
	12000 /10 H_2SO_4	2422.9		
ANF/COF	500 /10 H_2SO_4	250.3	1×10^{-4}	[24]
	300 /10 H_2SO_4	358.6	1×10^{-4}	
CPT	500 /1 HCl	46.2	0.03	[19]
	500 /10 HCl	37.8	0.03	
